# Combined physiological, transcriptome, and cis-regulatory element analyses indicate that key aspects of ripening, metabolism, and transcriptional program in grapes (*Vitis vinifera* L.) are differentially modulated accordingly to fruit size

**DOI:** 10.1186/s12864-016-2660-z

**Published:** 2016-05-31

**Authors:** D. C. J. Wong, R. Lopez Gutierrez, N. Dimopoulos, G. A. Gambetta, S. D. Castellarin

**Affiliations:** Wine Research Centre, University of British Columbia, Vancouver, BC Canada; Bordeaux Sciences Agro, Institut des Sciences de la Vigne et du Vin, Ecophysiologie et Génomique Fonctionnelle de la Vigne, UMR 1287, F-33140 Villenave d’ Ornon, France

**Keywords:** Aroma, Cell wall, Flavonoid, Grapevine, Hormone, Promoter, Quality, RNA-seq, Secondary metabolism, Transcriptomics

## Abstract

**Background:**

In wine grape production, management practices have been adopted to optimize grape and wine quality attributes by producing, or screening for, berries of smaller size. Fruit size and composition are influenced by numerous factors that include both internal (e.g. berry hormone metabolism) and external (e.g. environment and cultural practices) factors. Combined physiological, biochemical, and transcriptome analyses were performed to improve our current understanding of metabolic and transcriptional pathways related to berry ripening and composition in berries of different sizes.

**Results:**

The comparison of berry physiology between *small* and *large* berries throughout development (from 31 to 121 days after anthesis, DAA) revealed significant differences in firmness, the rate of softening, and sugar accumulation at specific developmental stages. *Small* berries had significantly higher skin to berry weight ratio, lower number of seeds per berry, and higher anthocyanin concentration compared to *large* berries. RNA-sequencing analyses of berry skins at 47, 74, 103, and 121 DAA revealed a total of 3482 differentially expressed genes between *small* and *large* berries. Abscisic acid, auxin, and ethylene hormone pathway genes were differentially modulated between berry sizes. Fatty acid degradation and stilbenoid pathway genes were upregulated at 47 DAA while cell wall degrading and modification genes were downregulated at 74 DAA in *small* compared to *large* berries. In the late ripening stage, concerted upregulation of the general phenylpropanoid and stilbenoid pathway genes and downregulation of flavonoid pathway genes were observed in skins of *small* compared to *large* berries. Cis-regulatory element analysis of differentially expressed hormone, fruit texture, flavor, and aroma genes revealed an enrichment of specific regulatory motifs related to bZIP, bHLH, AP2/ERF, NAC, MYB, and MADS-box transcription factors.

**Conclusions:**

The study demonstrates that physiological and compositional differences between berries of different sizes parallel transcriptome changes that involve fruit texture, flavor, and aroma pathways. These results suggest that, in addition to direct effects brought about by differences in size, key aspects involved in the regulation of ripening likely contribute to different quality profiles between *small* and *large* berries.

**Electronic supplementary material:**

The online version of this article (doi:10.1186/s12864-016-2660-z) contains supplementary material, which is available to authorized users.

## Background

Grapes (*Vitis vinifera* L.) are a highly valued horticultural crop with production covering approximately 7 million ha in 90 countries. The grape berry is extremely rich in secondary metabolites ranging from anthocyanins, carotenoids, norisoprenoids, tannins, terpenes, and other volatile organic compounds. These metabolites are very important to wine production as they affect wine quality by determining its color, aroma, and flavor [1]. Wine-grape growers adopt various vineyard management practices in order to optimize grape and wine quality attributes. Some of these practices involve producing (e.g. via applied water deficits) and/or selecting (e.g. via postharvest sorting) berries according to size with the belief that better wines are produced from small berries due to a higher skin to berry weight ratio. The reasoning being that this higher skin to berry weight ratio results in higher concentrations of key secondary metabolites accumulated in the skin [2, 3]. These practices are becoming increasingly popular and machines are even sold to automatically sort berries based on size.

The grape berry is a non-climacteric fruit with a double-sigmoidal growth curve that can be separated into three major stages [4]. The first stage of development sees a rapid increase in berry size due to high rates of cell division and expansion in the berry pericarp. Coinciding with the rapid growth is the biosynthesis of phenolic compounds, such as tannins and hydroxycinnamates, and organic acids, such as tartaric and malic acid. In the second stage, the berry experiences a lag phase, where pericarp growth is arrested and the embryo completes its development. At the end of the second stage, the berry undergoes veraison (the onset of ripening) and enters the third and final stage. During this phase, the berry experiences a second period of rapid cell expansion as the pericarp grows to its final size. Many changes in berry metabolism occur: accumulation of sugars, decrease in organic acid concentration, and production of various secondary metabolites. Thus, berry size and composition will differ depending on the stage of development.

Hormones play central roles in berry ripening and as environmental mediators (reviewed in [5]). During the first phase of development, auxins are present at high levels, and then decrease to very low levels as the berry undergoes veraison [6, 7]. Just prior to veraison, a small transient peak in ethylene is observed [8, 9] as well as sharp increases in abscisic acid (ABA) [10–13]. These observations indicate that ABA and ethylene are strong candidates as promoters of ripening. Treating berries with these hormones can affect the timing of veraison as well as other parameters, including fruit size and composition (reviewed in [5]). For example, treating berries with auxin prior to veraison temporarily delays berry growth, and sugar and anthocyanin accumulation [6].

Many factors influencing berry size are intrinsic, being related to the individual berry itself, such as seed number [14] and seed weight [15]. Recent evidence has shown that Pinot Noir and Cabernet Sauvignon seeded berries showed greater cell division and expansion than unseeded ones, resulting in larger berries [16]. Gouthu and Deluc [17] found a relationship between seed relative abundance and hormone content, where there were distinct differences in auxin and ABA levels between berries with different seed to berry weight ratios prior to veraison.

External factors, such as the environment and cultural practices, can also play a remarkable role in determining berry size. Water deficit [18, 19] and some pruning strategies [20] can result in smaller berries with higher phenolic content in the skin. However, other treatments may lower the concentration of some phenolics along with a reduction in berry size. This is seen with differences in the microclimate of clusters, where shading results in smaller berries with a lower phenolic content in the skin compared to light exposed clusters [21].

There are contrasting conclusions regarding differences in berry composition when comparing berries strictly on size. Some research indicates no differences between sizes [19, 22], while other research shows smaller Sauvignon Blanc berries having lower methoxypirazine concentrations (herbaceous aromatics accumulated in several cultivated varieties) compared to larger berries [23]. Similarly, there is no consensus on whether smaller berries make superior wines. Gil et al. [24] demonstrated that smaller grapes produced wines of deeper color and that size is inversely correlated with the concentration of phenolics, such as anthocyanins and stilbenes. In contrast, others have found that smaller berries do not translate into discernable differences in the resulting wines [22].

Changes in gene expression often parallel changes in berry skin metabolism throughout berry development [12, 25–28] and in response to stresses, such as water deficit [12, 29] and UV [30]. Many transcripts related to berry ripening and quality traits, such as those involved in hormone, phenylpropanoid, terpenoid, fatty acid, and cell wall metabolism, are modulated during development and in response to the environment. However, despite the strong impact on wine composition and quality, the regulation of transcripts/pathways related to ripening and berry composition in skins of differently sized berries is still unknown. In this study, our goal was to better understand the relationship between berry size, fruit ripening, and berry metabolism. We characterized how field grown Merlot berries differ in their ripening, metabolism, and transcriptional program in relation to berry size. We considered two extreme size classes and compared berry physiology, composition, and whole genome gene expression (RNA-sequencing) in the skin throughout development in order to determine the biological processes that discriminate between the two classes of berries.

## Methods

### Sample collection and physiological measurements

*V. vinifera* L.’Merlot’ clone 181 vines were grown in a vineyard located in Oliver (British Columbia, Canada; 49°13′18.8″N 119°33′28.8″W). Three biological replicates of fruit samples were harvested from three separate rows. Each replicate had 120 to 170 berries randomly collected from 30 vines per row at 31, 39, 47, 51, 54, 57, 60, 64, 67, 74, 103, and 121 days after anthesis (DAA) in 2014. Care was taken to avoid physical damage as berries were trimmed off the cluster at the pedicel and placed into aluminized mylar zip-lock bags to prevent water and turgor loss [31]. The bags were then immediately placed into a cooling box at ambient temperature and transported to the laboratory. Berry diameter and elasticity (E), a precise quantification of berry firmness [13], were recorded for each berry [32] and berries were individually bagged, labeled, and stored at −80 °C until the following analyses were conducted. From the population of berries at each sampling point, for each biological replicate seven berries were selected and pooled according to their size. Two pools were created: *large* berries, consisting of berries included in the top 90–95th percentile and *small* berries, consisting of berries included in the bottom 10–15th. These berries were used to calculate the evolution of E and total soluble solids (TSS), using a manual refractometer, across the season. Additional pools of seven berries selected from the same percentile at 47 (before ripening, 4.9 °Brix), 74 (early ripening, 17.5 °Brix), 103 (ripening, 22.4 °Brix) and 121 (late ripening, 25.3 °Brix) were used for both metabolite (see following paragraphs) and RNA-sequencing (RNA-seq) analyses. Three biological replicates for each treatment were considered for both metabolite and RNA-seq analysis.

### Liquid chromatography (LC) and LC-mass spectrophotometric (MS) analysis

Berries were peeled while still frozen using a scalpel. Flesh tissue was then used for determining sugar and organic acid concentration, and skin tissue for anthocyanin content.

For the determination of sugar (glucose and fructose) and organic acid (malic and tartaric acid) content, flesh tissues were grounded to a fine powder and centrifuged at 16,000 g for 10 min. Afterwards, the supernatant was passed through a 0.22 μm PVDF 13 mm filter (Whatman Inc., Sanford, USA) and measured via HPLC on an Agilent 1100 HPLC system with a refractive index detector (Agilent Technologies, USA). An aliquot of 20 μl of extract was injected into a NUCLEOGEL® ION 300 OA column (300 mm × 7.8 mm ID, 10 μm) (Machery-Nagel Inc., USA), maintained at 71 °C. The mobile phase was 2.5 mM H_2_SO_4_ with a flow rate of 0.55 ml/min. Quantification of metabolite concentration (expressed as mg/ml of juice) was based on calibration curves of authentic standards.

For the extraction of anthocyanins, 0.2 g of skins were grounded and extracted in 2 ml 50 % (*v/v*) methanol in water for 3 h with rigorous shaking. The supernatant was centrifuged, filtered and measured on an Agilent 1100 LC/MSD Trap XCT Plus mass spectrometer. Separation of anthocyanin compounds was achieved on an Agilent Zorbax SB-C18 column (150 mm × 4.6 mm ID, 1.8 μm) held at 57 °C. The mobile phases used were: water-formic acid (2 %), solvent A, and acetonitrile-formic acid (2 %), solvent B. Flow rate was 0.8 ml/min. The solvent gradient program was 0.5 min, 6 % B; 4 min, 10 % B; 13 min, 25 % B; 20 min, 35 % B; 25 min, 60 % B; 30 min, 95 % B; and 32 min, 6 % B. The anthocyanins mass spectra were analyzed after electrospray ionization (ESI) in alternating positive and negative ionization mode with a scan range between 50 and 850 m/z. Quantification of single and total anthocyanins was based on malvidin 3-glucoside equivalents (expressed as mg/g fresh weight of berry skin). The percentages of 3′4′-OH, 3′4′5′-OH, and methoxylated anthocyanins were calculated among monoglucoside anthocyanins.

### RNA extraction, sequencing, and data analysis

Extraction of total RNA was achieved with the Spectrum Plant Total RNA kit (Sigma-Aldrich) using ~80 mg of grounded skins according to the manufacturer’s protocol. The integrity of the extracted RNA was determined on an Agilent 2100 Bioanalyzer (Agilent) ensuring a RIN score >7.5 prior to library construction. Ribosomal RNA depleted library construction was performed using an in-house workflow using customized kits from NEB at the Canada’s Michael Smith Genome Sciences Center (Vancouver, Canada) followed by sequencing (V4 chemistry) on an Illumina HiSeq 2500 platform (Illumina) and in-house quality control and filtering of 75-bp paired-end generated reads. Filtered pair-end reads were aligned against the 12× reference genome [33] using Burrows-Wheeler Aligner with default parameters [34]. Read summarization was performed with htseq-count (version 0.6.0) with intersection non-empty settings [35] using the grapevine 12× genome assembly available from EnsemblPlants (http://plants.ensembl.org/Vitis_vinifera/Info/Index). Differential gene expression analysis was carried out using edgeR (version 3.10.2) [36]. Genes were deemed differentially expressed (DE) between pairwise comparisons at a threshold of false discovery rate (FDR) < 0.05. Transcript abundance was calculated as Fragments Per Kilobase of exon per Million (FPKM) mapped reads using edgeR. Transcripts having FPKM values <0.5 and assigned counts <5 were discarded.

### Clustering, functional enrichment, and promoter analysis

Clustering of DE genes using edgeR’s estimated gene expression (normalized counts) log2 fold change (log2FC) between *small*/*large* berries was performed using the k-means method with 1000 iterations and the Speaman’s rank correlation as the similarity metric. The latest grapevine gene annotations based the 12× V1 models were obtained from [37]. A separate functional annotation of transcripts was performed using the Mercator pipeline [38] to ascribe potential gene function and MapMan BINs [39] prior to gene enrichment analysis. MapMan BIN categories were considered significantly enriched (adjusted *P*-value <0.05) as determined by Fisher’s exact test adjusted with Bonferroni correction for multiple testing correction. Based on the 12× grapevine genome assembly, all grapevine promoter sequences (1 kb upstream of the 5′ UTR) were retrieved from the CRIBI grape genome database (http://genomes.cribi.unipd.it/grape/) [40]. Target sequences (cis-regulatory elements, CREs) of 63 plant transcription factors (TFs) representing 25 families recently characterized in *Arabidopsis thaliana* [41] and the ones from PLACE [42] were retrieved. These sequences were scanned in promoter regions of DE genes within clusters and selected gene families. Motif overrepresentation was calculated according to [43] using hypergeometric test and adjusted with false discovery rate (FDR) for multiple testing correction when appropriate. Putative CREs was considered significantly enriched if the associated FDR < 0.01.

### Statistical analysis

A one-way ANOVA was performed using JMP 7 (SAS Institute Inc.) to detect significant differences (*P* < 0.05, *P* < 0.01, *P* < 0.001) in berry components, E, TSS, and sugar, organic acid, and anthocyanin levels between *small* and *large* berries treatments at each sampling point.

## Results and discussion

### Analysis of physiological and compositional parameters between *small* and *large* berries

**Fig. 1:**
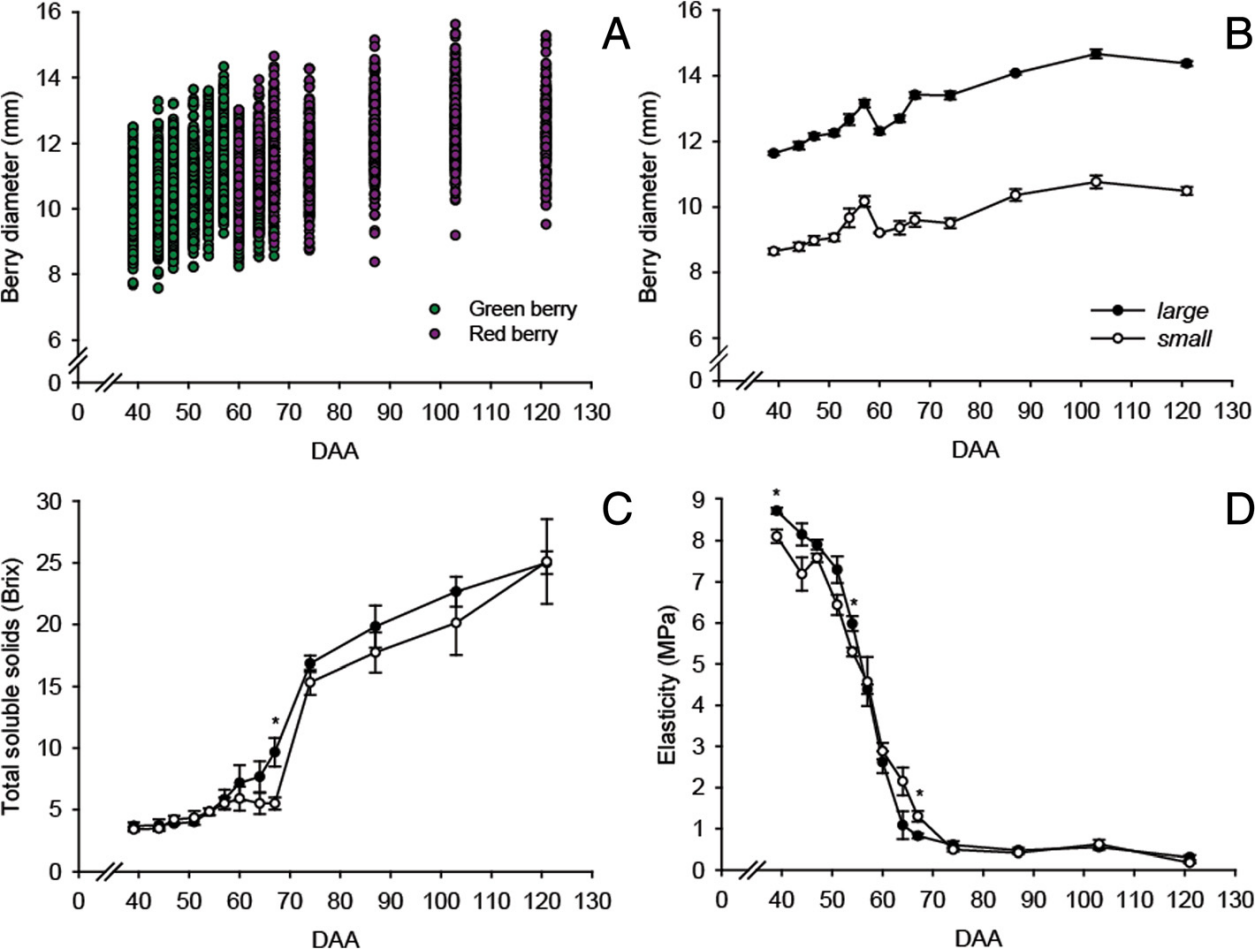
Evolution of berry diameter, total soluble solids, and elasticity. (**a**) Berry diameter of the entire sampled population. (**b**) Diameter, (**c**) total soluble solids, and (**d**) elasticity of *small* and *large* berries during development. *Green* and *purple* indicates the recorded color of each individual berry. Means and standard errors are reported for each berry group at each developmental stage. * indicates a significant difference (*P* < 0.05) between *small* and *large* berries

The diameters of all the berries considered in the experiment are reported in Fig. 1a. Berry diameter, total soluble solids (TSS, °Brix), and elasticity (E) evolution in *small* and *large* berries during fruit development are reported in Fig. 1b, c, and d. Despite the differences in absolute values, the evolution of berry diameter was similar between *small* and *large* berries throughout the season (Fig. 1b). The TSS levels of *small* and *large* berries increased from 51 DAA until harvest (Fig. 1c). A faster increase in TSS was observed for *large* berries from 60 to 67 DAA. TSS levels of *small* berries remained relatively low until 67 DAA; afterward, a sharp increase was observed. E measures berry firmness, so a firm berry has higher E values (i.e. more pressure is required for a particular displacement), and as a berry softens E decreases [13, 31]. E of the *small* and *large* berries strongly decreased from 47 to 74 DAA, afterward the decrease was gradual (Fig. 1d). Differences in E levels and in the rate of E decrease between *small* and *large* berries were apparent at certain developmental stages. For example, at 39 and 54 DAA, *large* berries had higher E than *small* berries, while between 60 and 74 DAA, *small* berries had higher E than *large* ones. Although no significant differences in E levels were observed at 54 and 57 DAA, a steeper drop of E was observed in *large* than in *small* berries from 54 to 74 DAA (Fig. 1d).

**Fig. 2:**
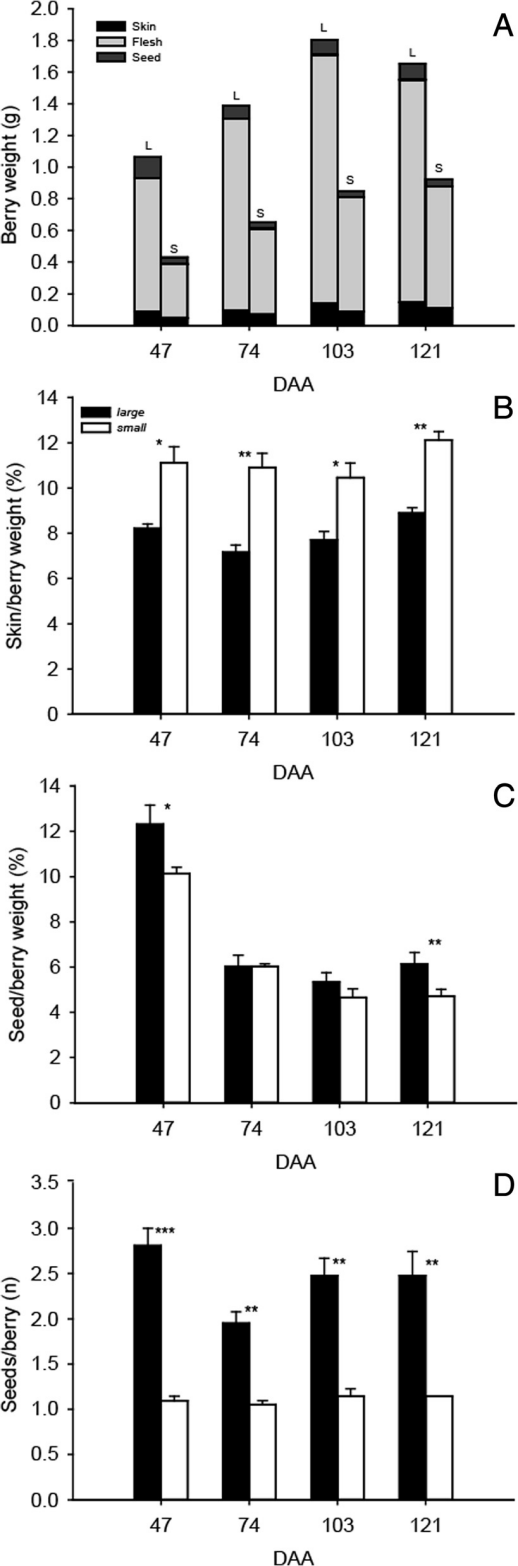
Berry features. (**a**) Skin, flesh, and seed weight, (**b**) skin to berry weight ratio, (**c**) seed/berry weight ratio, and (**d**) seeds/berry number in *small* and *large* berries at 47, 74, 103, and 121 DAA. *, **, and *** indicate level of significance of *P* < 0.05, *P* < 0.01, and *P* < 0.001, respectively

**Fig. 3:**
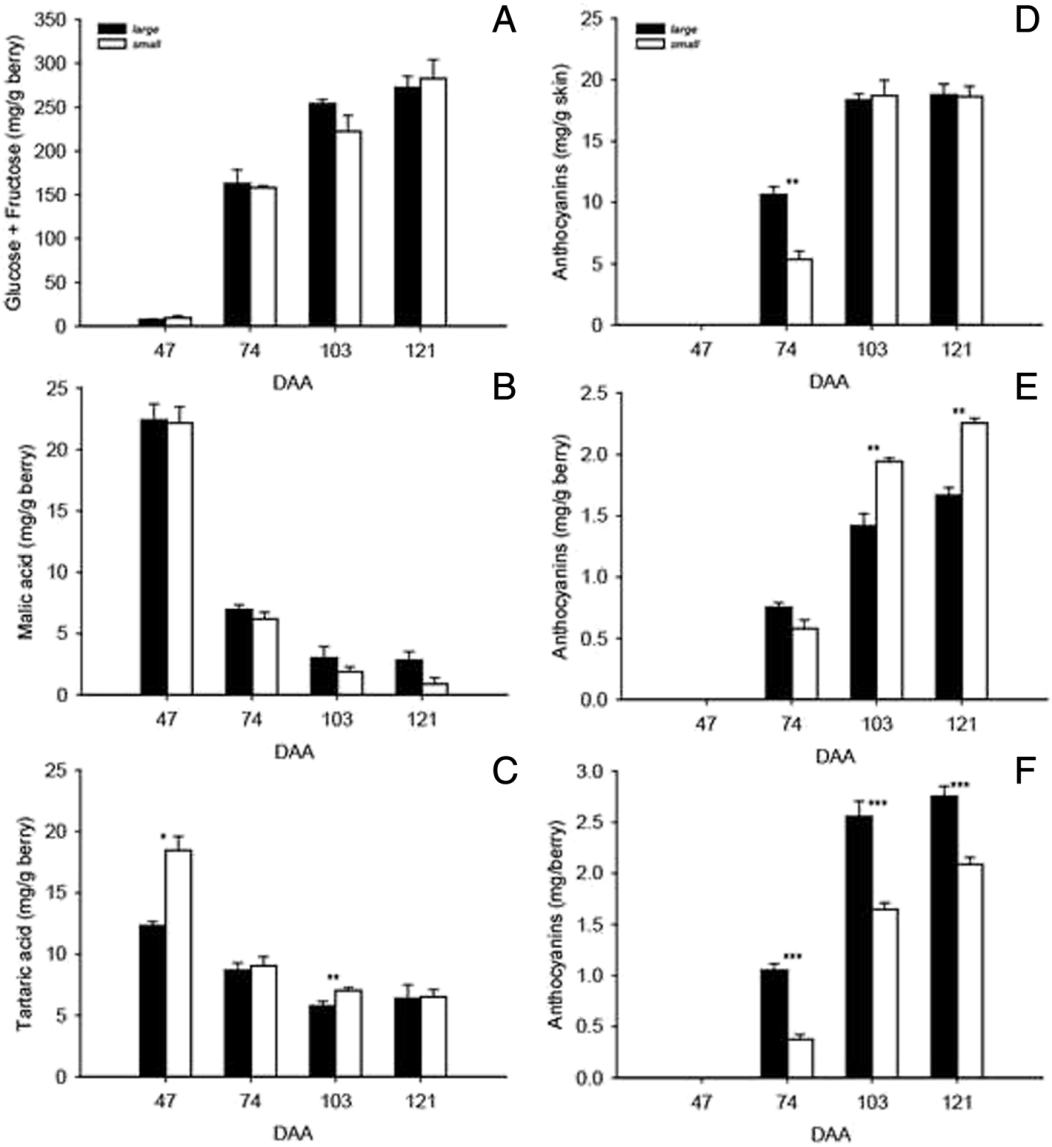
Berry composition. (**a**) Glucose + fructose, (**b**) malic acid, and (**c**) tartaric acid concentration, expressed as mg/g berry, in *small* and *large* berries at 47, 74, 103, and 121 DAA. Anthocyanin levels expressed as (**d**) mg/g skin, (**e**) mg/g berry, and (**f**) mg/berry in *small* and *large* berries at 47, 74, 103, and 121 DAA. Means and standard errors are reported for each berry group at each sampling. *, **, and *** indicate level of significance of *P* < 0.05, *P* < 0.01, and *P* < 0.001, respectively

Berries from 47, 74, 103, and 121 DAA were selected for further physiological and biochemical analysis (Figs. 2 and 3). The skin to berry weight ratio was significantly higher in *small* than *large* berries at all four developmental stages (Fig. 2b); while *large* berries had significantly higher seed to berry weight ratios at 47 and 121 DAA (Fig. 2c). In addition, significant differences in the number of seeds/berry were observed between sizes, where *large* berries have more seeds (Fig. 2d), consistent with previous studies [15, 22].

Glucose and fructose levels increased sharply from 47 to 74 DAA and continue to increase until 121 DAA (Fig. 3a). Malic and tartaric acids (Fig. 3b and c) were at high levels at 47 DAA, and progressively decreased from 74 DAA onwards. The levels of sugars and organic acids reported here are comparable with the levels found in different cultivars at the same developmental stage [12, 21, 44]. No significant differences in the concentrations of sugars, glucose and fructose (Fig. 3a), and malic acid (Fig. 3b) were observed between *small* and *large* berries for any of the sampling dates. Tartaric acid concentration was significantly higher in *small* than in *large* berries at 47 and 103 DAA (Fig. 3c).

The total anthocyanin content (mg/g of skin) was significantly lower in *small* berries at 74 DAA (Fig. 3d). Although the recorded veraison (50 % of the berries display red pigmentation) date in the vineyard was 60 DAA, the large increase in sugar levels in *large* berries prior to 70 DAA (Fig. 1c) could have stimulated an earlier accumulation of anthocyanin in these berries [45, 46]. The accelerated drop in E observed in *large* berries, as well as the faster increase in sugar and anthocyanin levels, suggest that *large* berries approach ripening faster than *small* berries. Although the anthocyanin concentration in skins (mg/g skin) was similar between *small* and *large* berries at 103 and 121 DAA (Fig. 3d), *small* berries had a significantly higher anthocyanin concentration expressed as mg/g of berry (Fig. 3e). Vice versa, total anthocyanin content, expressed as mg/berry, was higher in *large* berries (Fig. 3f). This suggests that the increase in anthocyanin concentration observed in *small* berries at 103 and 121 DAA was not due to a higher synthesis of these pigments but to the higher skin to berry weight ratio (Fig. 2b). This higher proportion of skin tissue determines the higher concentration of anthocyanin in *small* berries as observed in [19].

### Analysis of transcript differences between *small* and *large* berries

To determine the influence of berry size on the berry skin transcriptome, Illumina mRNA sequencing was performed on berry skins at the four selected developmental stages (47, 74, 103, and 121 DAA). Using the 12× V1 genome as reference [33], an average of 34 million high-quality paired-end reads per sample were successfully mapped, which typically corresponds to 92.5 % of paired-end reads for each library (Additional file 1: Table S1). An average of 29 million paired-end reads per sample was assigned to transcripts. We detected the presence of 23,012 unique transcripts expressed in at least one of four developmental stages. Approximately 18,600, 17,800, 17,200, and 17,150 transcripts were expressed in berry skins at 47, 74, 103, and 121 DAA, respectively.

**Fig. 4:**
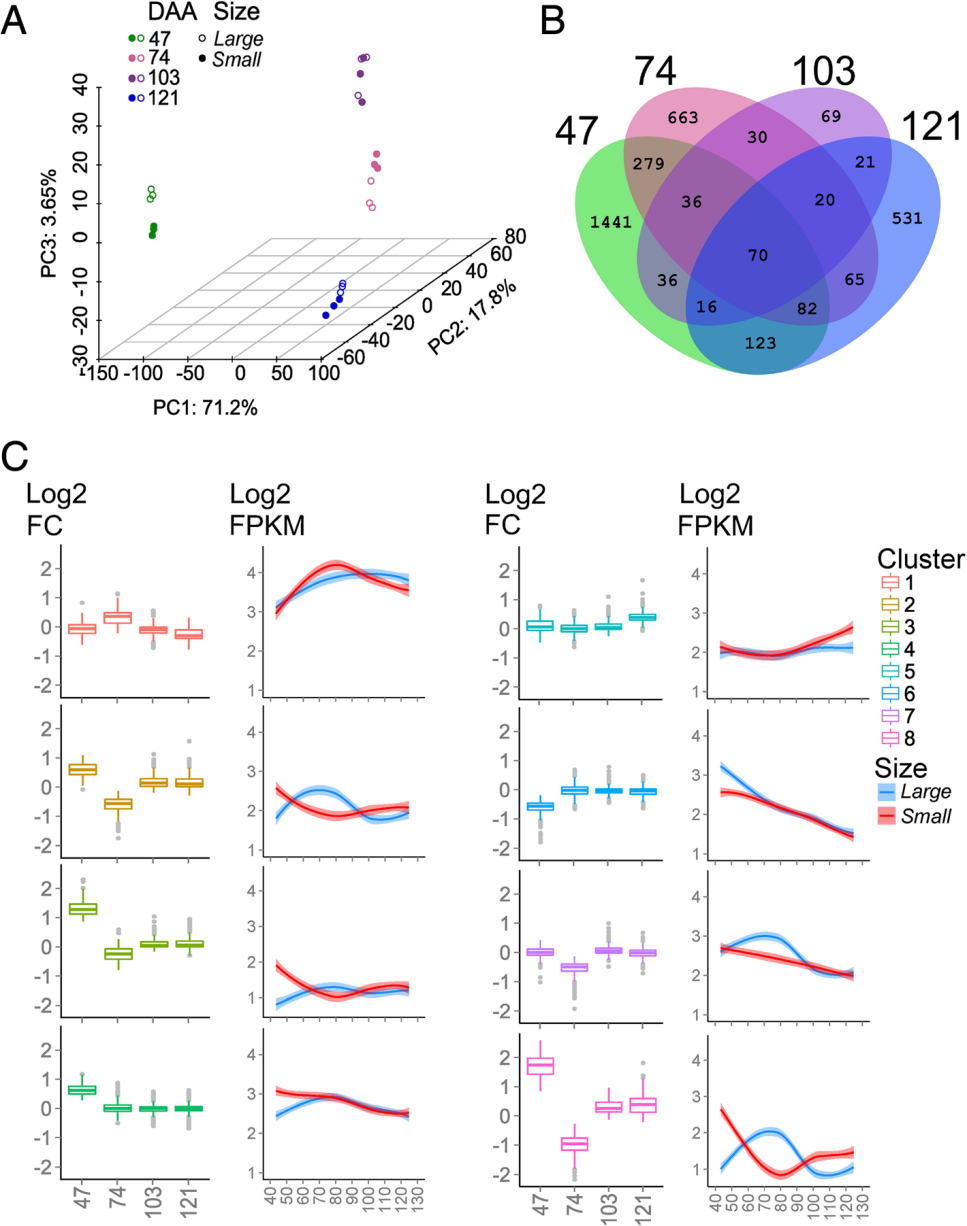
Analysis of the berry skin transcriptome of *small* and *large* berries. (**a**) Principal component analysis (PCA) of the berry skin transcriptome of *small* (*filled circles*) and *large* (*empty circles*) berries at 47 (*green*), 74 (*pink*), 103 (*purple*), and 121 (*blue*) DAA. (**b**) The Venn diagram represents the common and unique genes differentially expressed between *small* and *large* berries at 47, 74, 103, and 121 DAA. Differentially expressed genes in each intersection of the Venn diagram are described in Additional file 1: Table S3. (**c**) The *box plot* and *smoothed line plot* represents the response of *small* versus *large* berries and dynamic change of gene expression during berry development, respectively. Differentially expressed genes were clustered using the k-means algorithm. The log2 fold changes between *small *and *large* and log2 (FPKM +1) values at 47, 74, 103, and 121 DAA were used. Outlier log2 fold change values are represented as *grey circles*

A principal component analysis (PCA) was undertaken to analyze the level of similarity of the transcriptomes analyzed. The first three principal components explained a cumulative variance of 92.7 %; with the first, second, and third principal component explaining 71.2, 17.8, 3.7 % of the variance, respectively (Fig. 4a). Inspection of the PCA plots revealed a clear separation of berry skin transcriptome based on the developmental stage and not on the biological variation within a developmental stage. Furthermore, a separation of the transcriptomes driven by the berry size was more evident at 47, 74, and 121 DAA, while it was undetected at 103 DAA. A total of 3482 unique genes (11.6 % of predicted transcriptome) were identified to be differentially (FDR < 0.05) expressed between *small* and *large* berries in at least one developmental stage (Additional file 1: Table S2 and S3). A total of 2083 (557 downregulated, 1526 upregulated), 1244 (983 downregulated, 261 upregulated), 298 (57 downregulated, 241 upregulated) and 928 (375 downregulated, 573 upregulated) were differentially expressed (DE) between *small* and *large* berries at 47, 74, 103, and 121 DAA, respectively (Fig. 4b). Gouthu et al. [47] demonstrated that differences in the ripening program (i.e. asynchrony) was reflected at the transcript level with large differential expression of berry transcripts when comparing between berry classes differing in softness and coloration at mid-veraison (~69 DAA), regardless of tissue. These differences were drastically reduced to near zero at maturity. Our study revealed that differences in the skin transcriptomes between *small* and *large* berries are larger at earlier stages of development (47 and 74 DAA) than at later stages of development (103 and 121 DAA). This difference, together with the differences in fruit physiology and composition observed at the corresponding stages further suggests an asynchrony between *small* and *large* berries at early stages of development (Figs. 1a, d and 3c, d). The reduction of DE (from 47 to 103 DAA) suggests a process of resynchronization of the transcriptome between *small* and *large* berries during development. Interestingly, there was a second increase of the number of DE transcripts at 121 DAA. This might reflect the activation of an over-ripening/senescence program in the skins of *small* berries [28]. Cramer et al. [28] investigated the evolution of the skin transcriptome at late developmental stages, including over-ripening stages. Approximately 15 % of the top 1000 DE genes were the same when results were compared between the DE genes between *small* and *large* berries at 121 DAA (25 °Brix) in this study and between 25 and 38 °Brix in Cramer et al. [28], suggesting that *small* berries are ahead in ripening at these later stages.

Differentially expressed genes were clustered into 8 clusters based on their log2 fold change differences in *small* with respect to *large* berries at each development stage using the k-means clustering (Fig. 4c). Functional annotation of DE genes according to high-level MapMan ontology categories [39] showed that the largest proportion was involved in RNA regulation and protein metabolism (8–10 %), while transport, cell wall, signaling, and primary and secondary metabolism categories contributed between 5 and 8 % of the total DE genes (Table 1, Additional file 1: Table S3). A total of 382 (of ~2213) transcripts annotated as potential grapevine transcriptional regulators [37] were DE between *small* and *large* berries within each sampling date (Additional file 1: Table S3), indicating a difference in transcript regulatory programs between *small* and *large* berries. Validating the observation that a large number of TF were DE between *small* and *large* berries, many cis-regulatory elements (CREs) related to binding sites of various TF families were also enriched among the DE genes (Table2, Additional file 1: Table S4), which may play an important role in the regulation of DE genes. The frequent occurrences of multiple enriched CREs within promoter regions of DE transcripts will be discussed further in the context of metabolic pathway regulation (see below).

**Table 1:** Enrichment of MapMan functional categories (BINs) of k-means assigned clusters containing differentially expressed transcripts comparing skins of *large* and *small* berries. The table contains detailed information of the cluster size, BIN code and associated description, and the Bonferroni-adjusted *P*-value (Adj. *P*-value <0.05). Only high-level BINs are presented in this table (up to one decimal or depth = 1)

Cluster	Size	BIN	BIN name	Adj. *P*-value
1	472	16	secondary metabolism	0.000476
		16.8	secondary metabolism.flavonoids	2.78E-07
		18.2	Co-factor and vitamine metabolism.thiamine	0.043163
		20.2	stress.abiotic	0.005947
		33.1	development.storage proteins	0.002419
2	342	5.3	fermentation.ADH	0.00028
3	265	NA	NA	NA
4	884	10.7	cell wall.modification	0.00655
		27	RNA	0.008355
		27.3	RNA.regulation of transcription	2.15E-07
		30	signalling	0.043608
		30.3	signalling.calcium	7.64E-05
5	320	16	secondary metabolism	1.54E-07
		16.8	secondary metabolism.flavonoids	2.81E-08
6	553	10	cell wall	8.42E-10
		10.8	cell wall.pectin*esterases	0.002015
		26	misc	0.001691
		26.28	misc.GDSL-motif lipase	0.014839
		30	signalling	0.001752
		30.2	signalling.receptor kinases	0.000168
7	480	10	cell wall	0.002828
		10.6	cell wall.degradation	0.033172
		17	hormone metabolism	7.42E-05
		17.1	hormone metabolism.abscisic acid	1.1E-08
		20.2	stress.abiotic	0.002289
		34	transport	0.025226
		34.4	transport.nitrate	0.024422
8	166	15	metal handling	0.00843
		33	development	0.041594
		33.99	development.unspecified	0.012587
		34	transport	0.005185
		34.12	transport.metal	0.024644

**Table 2:** Enrichment of PLACE- and PBM-curated cis-regulatory elements (CRE) of selected k-means assigned clusters containing differentially expressed transcripts comparing skins of *small* and *large* berries. The table contains detailed information on the number of promoters with the specified CRE, number of promoters in the genome containing the specified CRE, CRE sequence, false discovery rate (FDR), and the designated CRE name and regulatory description. A full description on the corresponding genes containing the associated CRE is available in Additional file 1: Table S4

Cluster	Matches in sample	Matches in genome	FDR	Motif	Motif name	Regulation
1	92	3331	9.67E-06	ACGTGKC	ACGTABREMOTIFA2OSEM	ABRE
	146	6199	9.67E-06	CACGTG	CACGTGMOTIF	bHLH/bZIP
	98	4120	0.000552	AGATATTT	CCA1-1	MYB-related
	33	1008	0.0024	YACGTGGC	ABREATCONSENSUS	ABRE
	54	2115	0.004942	CACGTGG	IRO2OS	bHLH/bZIP
4	109	1808	1.27E-10	CACGCG	MYC2-5	bHLH
	397	10,397	7.44E-09	MACGYGB	ABRERATCAL	ABRE
	469	13,792	0.000232	GCCAC	SORLIP1AT	Light
	137	3331	0.000652	ACGTGKC	ACGTABREMOTIFA2OSEM	ABRE
	286	7976	0.000884	WTTSSCSS	E2FCONSENSUS	E2F
	227	6199	0.001528	CACGTG	CACGTGMOTIF	bHLH/bZIP
	120	3057	0.005201	GAGTGAG	SORLIP5AT	Light
	303	8835	0.005201	CTGACY	WBOXNTCHN48	WRKY
5	154	10,397	9.78E-05	MACGYGB	ABRERATCAL	ABRE
6	131	4411	1.51E-06	TGTCGG	ETT-1	ARF
	149	5482	1.95E-05	ACCGAC	DRE2COREZMRAB17	AP2/ERF
	185	7231	1.95E-05	RCCGAC	DRECRTCOREAT	AP2/ERF
	285	12,862	0.000589	RYCGAC	CBFHV	AP2/ERF
	325	15,123	0.000887	CCWACC	MYBPZM	MYB
	248	11,172	0.002184	ACCWWCC	BOXLCOREDCPAL	MYB
	128	5288	0.006668	TGTCGA	ETT-2	ARF
	46	1504	0.0082	GGNCCCAC	TCP15	TCP
7	67	2312	0.000349	GCCGAC	RAP2.3-3	AP2/ERF
	153	7231	0.003952	RCCGAC	DRECRTCOREAT	AP2/ERF
	116	5288	0.005831	TGTCGA	ETT-2	ARF
	202	10,397	0.008081	MACGYGB	ABRERATCAL	bZIP/bHLH
	241	12,862	0.008373	RYCGAC	CBFHV	AP2/ERF

Because of the observed differences between *small* and *large* berries at the physiological level and of the potential role of some of the DE genes on determining fruit and wine quality, we focused our discussion on pathways related to hormone metabolism and signaling, cell wall modifications, and flavonoid, stilbenoid, and fatty acid biosynthesis.

### Modulation of hormone metabolism and signaling in *small* and *large* berries

**Fig. 5:**
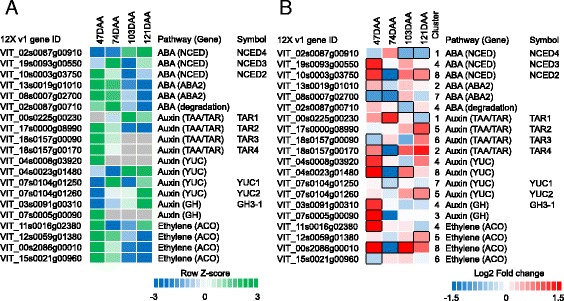
Evolution during development and fold change between *small* and *large* berries of abscisic acid (ABA), auxin, and ethylene genes differentially expressed at 47, 74, 103, and 121 DAA. (**a**) Evolution, based on the mean log2 (FPKM + 1) of *small* and *large* berries, and (**b**) log2 fold (*small/large*) changes. The relative log2 (FPKM + 1) values registered in *small* and *large* berries on average during berry development in **a** are depicted by *green* (high expression) and *blue* (low expression). *Grey color* indicates the absence (or low levels) of detectable transcripts at the corresponding stage. *Blue* and *red* colors in **b** indicate downregulated and upregulated transcripts, respectively, in *small* berries in relation to *large* berries. *Boxes* with *bold margins* indicate significant differences (adjusted *P*-value <0.05) between berry size treatments at a given developmental stage. The cluster column in **b** indicates the cluster number the associated transcript belongs to NCED, 9-cis-epoxycarotenoid dioxygenase; ABA2, xanthoxin dehydrogenase; TAA/TAR, TRYPTOPHAN AMINOTRANSFERASE OF ARABIDOPSIS1/TRYPTOPHAN AMINOTRANSFERASE RELATED (TAA1/TAR); YUC, YUCCA; GH, IAA-amido synthetase; ACO, 1-aminocyclopropane-1-carboxylic acid oxidase

Regulation of fruit development and ripening involves major metabolic changes regulated by complex interactions among hormones and not by a single hormone in isolation [48]. In grapes, ABA promotes berry ripening and auxins delay this process, while results on ethylene are mixed [5]. One hundred fifty-seven genes annotated to function in hormone metabolism (i.e. biosynthesis, degradation, signaling and transcriptional regulation; BIN 17) were DE between *small* and *large* berries (Additional file 1: Table S5). A total of 38, 42, and 29 transcripts related to ABA, auxin, and ethylene metabolism and signaling, respectively, were the most represented among hormone related genes, followed by 18, 14, 11, and 6 transcripts related to jasmonic/salicylic acid, cytokinin, brassinosteroid, and gibberellin metabolism and signaling, respectively. Due to their major role in the ripening process [5], emphasis will be given to the transcripts involved in the biosynthetic pathways of ABA, auxin, and ethylene (Fig. 5).

Transcripts encoding structural enzymes of the ABA biosynthetic pathway were modulated during fruit development and were DE between *small* and *large* berries (Fig. 5a and b). Transcripts encoding 9-cis epoxycarotenoid dioxygenase (NCED) regulate ABA biosynthesis and the alteration of NCED expression have direct implications in ABA concentration and ripening-related traits in fruits [49]. The expression patterns of three DE *NCED* transcripts in the skin were distinct (Fig. 5a). For example, *NCED2* (VIT_10s0003g03750) transcripts peak at 47 DAA and gradually decrease towards 121 DAA, *NCED3* (VIT_19s0093g00550) transcripts peak at 74 DAA and again at 121 DAA, while *NCED4* (VIT_02s0087g00910) transcripts progressively increase towards 121 DAA. These observations were consistent with previous studies showing that *NCED* transcripts are under complex regulation in the berry [10–12, 50] and that expression of *NCED*s is required to maintain in situ berry ABA biosynthesis. Several studies have highlighted that *NCED3* is the enzymatic isoform which is correlated with berry ABA accumulation [9, 11]. Therefore, the upregulation of this gene in *small* berries at 47 DAA may contribute to higher ABA levels at this stage; however, later during ripening when ABA levels are still high [11, 13, 47] no significant difference in *NCED3* transcripts was observed in the skins of *small* and *large* berries. Instead, *NCED2* transcripts were highly downregulated at 74 DAA in skins of *small* with respect to *large* berries. This might have contributed to differences in overall ABA accumulation and lower ABA accumulation in *small* berries at early stages of ripening would be consistent with the delay in sugar and anthocyanin accumulation observed at 67 and 74 DAA, respectively (Figs. 1 and 2). In addition, the downregulation of ripening-associated* NCED4* gene in *small *compared to *large *berries from 103 DAA onwards might also reflect the lower ABA biosynthetic capacity of *small* compared to *large* berries at 103 and 121 DAA. Nevertheless, *NCED2* was observed to be upregulated in *small* compared to *large* berries at 121 DAA. Collectively, different sized berries exhibit a different regulation of *NCED* transcripts, and significantly lower *NCED* transcripts levels were found in *small* berries than in *large* ones during berry ripening, especially at 74 DAA, with potential effects on the ripening process.

The genes encoding TRYPTOPHAN AMINOTRANSFERASE OF ARABIDOPSIS1/TRYPTOPHAN AMINOTRANSFERASE RELATED (TAA1/TAR) and YUCCA (YUC) are critical for regulating auxin levels and ripening in young berries [7]. The expression of transcripts encoding TAA1/TAR1 – 4 and YUC1 throughout berry development (2 to 16 weeks post flowering) have also been reported in Shiraz berries [7]. These genes were modulated during fruit development and were also DE between *small* and *large* berries in this study (Fig. 5a and b). Three *TAA/TAR* transcripts (*TAR2*, *3*, and *4*) and another (*TAR1*) peaked in the skins of berries harvested at 47 and 103 DAA, respectively. One *YUC* transcript (VIT_04s0008g03920) was specifically present at 47 DAA, another *YUC* transcript (VIT_07s0104g01250) peaked around veraison (74 DAA), and other two *YUC*s (VIT_07s0104g01260 and VIT_04s0023g01480) were mostly present during late ripening. These observations were consistent with previous studies showing *TAA/TAR* and *YUC* transcript evolution and auxin accumulation during berry development and ripening [7]. At 47 DAA, when auxins levels are high, transcripts encoding TAR4 (VIT_18s0157g00170) and a YUC (VIT_04s0008g03920) were highly upregulated in *small* with respect to *large* berries. Similarly, *TAR1* (VIT_00s0225g00230) transcripts were also upregulated at 74 DAA. However, *TAR3* (VIT_18s0157g00090) transcripts, which have been shown to relate with the accumulation of auxin at early stages [6], were downregulated in *small* with respect to *large* berries at 47 DAA. Several studies have indicated that auxin conjugation through the action of IAA-amido synthetases (GH3), which conjugates aspartic acid to auxin, is critical for ripening initiation and the regulation of auxins in the berry during development [51, 52]. Interestingly, *GH3-1* transcripts (VIT_03s0091g00310) which display a ripening-associated developmental accumulation were significantly upregulated in the skins of *small* compared to *large* berries. Therefore, significant differences in ripening physiology of the two berry classes (Figs. 1, 2, and 3) and significant modulation of auxin metabolic pathways points to the possible involvement of auxin and its conjugates in determining asynchrony between berries of different sizes.

The biosynthesis of ethylene is determined, in part, by the action of 1-aminocyclopropane 1-carboxylate (ACC) oxidase (ACO), a key enzyme involved in the production of ethylene using ACC as precursor [48]. Several ACO-encoding members were also DE between *small* and *large* berries (Fig. 5b), many of which exhibit highest expression at 47 DAA followed by a gradual decrease until 103 DAA (Fig. 5a). Although grapevine is a non-climacteric fruit, previous findings have shown an increase of ACO activity and ethylene production at stages before veraison [8, 9]. Two *ACO* transcripts (VIT_11s0016g02380 and VIT_00s2086g00010) displayed strong upregulation at 47 DAA in *small* compared to *large* berries. This might provide evidence that in *small* berries at pre-veraison stages a higher production of ethylene may in fact be delaying the ripening process and not enhancing ripening, as exemplified by the delayed sugar accumulation at 68 DAA and the lower anthocyanin levels at 74 DAA in *small* berries (Fig. 3). Indeed, recent studies have demonstrated application of ethylene at pre-veraison stages delays ripening through the activation of auxin biosynthesis pathways, significantly postponing sugar and anthocyanin accumulation, as well as the berry growth [7, 53, 54]. Ethylene may also play a role later in ripening, especially in the skin, as the abundance of many transcripts involved in ethylene biosynthesis and signaling peaked from 23 °Brix onwards, stages when desirable flavor and aroma compounds accumulate [28]. In this study, the significant upregulation of two *ACO* transcripts (VIT_12s0059g01380 and VIT_00s2086g00010) from 103 DAA onwards, indicates that ethylene might contribute to the induction of pathways important for fruit flavor in the skin of *small* berries compared to *large* ones [28].

Among hormone related transcription factors, the grapevine bZIP TF, VvABF2, have been shown to modulate ABA-dependent berry ripening processes through the induction of cell wall hydrolytic enzymes and complex modulation of multiple hormonal pathways including ABA, auxin, and ethylene [55]. Expression of *VvABF2* transcript (VIT_18s0001g10450) was highest at 74 DAA corroborating its ripening-associated expression and its role in berry ripening (Additional file 1: Table S3). Interestingly, *VvABF2* was first upregulated in *small* berries at 47 DAA and subsequently downregulated at 74 DAA compared to *large* berries. This modulation might mediate the altered hormonal metabolism profiles involving *NCED*, *TAA/YUC*, and *ACO* transcripts between *small* and *large* berries at 47 and 74 DAA. Also, the regulation of cell wall degradation and expansion via ABF2 may explain the slower softening rate experienced in *small* compared to *large* berries observed at the onset of fruit ripening.

In summary, the differential regulation of several *NCED*, *TAA/YUC*, and *ACO* transcripts between the berry size classes suggest that hormonal pathways are differential regulated and might therefore contribute to variations in ABA, auxin, and ethylene levels in the skin of *small* and *large* berries with potential effects on skin metabolism and composition. However, additional studies with intensive sampling strategies and measurement of these hormones in the two berry classes will be needed to gain a deeper understanding on the relative contribution of each hormone and its influence on berry size and fruit composition traits.

### Modulation of cell wall metabolism in *small* and *large* berries

In fleshy fruits, the remodeling of cell wall architecture during fruit development affects fruit softening [56] and involves concerted changes in cell wall-related gene expression and multiple enzyme activities [48]. Influence of berry skins in determining post-veraison berry growth [57, 58] and the role of cell wall degradation and modification enzymes in mediating this process [59] have been previously reported. The altered transcriptional profiles of cell wall genes in skins between *small* and *large* berries highlights the potential role of cell wall modification genes in modulating berry growth and softening.

**Fig. 6:**
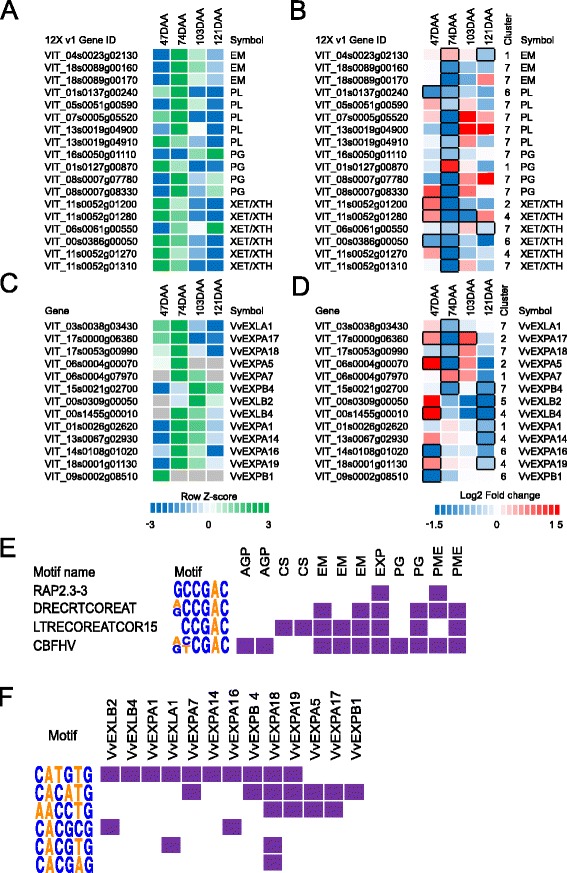
Evolution during development, fold change values between *small* and *large* berries, and selected cis-regulatory element profile of cell wall transcripts differentially expressed at 47, 74, 103, and 121 DAA. The heat map represents the evolution (**a** and **c**), based on the mean log2 (FPKM + 1) of *small* and *large* berries, and log2 fold (*small/large*) changes (**b** and **d**) of cell wall degradation (**a** and **b**) and modification (**c** and **d**) genes. The relative log2 (FPKM + 1) values registered in *small* and *large* berries on average during berry development in **a** and **c** are depicted by *green* (high expression) and *blue* (low expression). *Grey* color indicates the absence (or low levels) of detectable transcripts at the corresponding stage. *Blue* and *red boxes* in **b** and **d** indicate downregulated and upregulated transcripts, respectively, in *small* berries in relation to *large* berries. Boxes with bold margins indicate significant differences (adjusted *P*-value <0.05) between *small* and *large* berries at a given developmental stage. The cluster column in **b** and **d** indicates the cluster number the associated transcript belongs to. EM, 1,4-beta-mannan endohydrolase; PL pectate lyase; PG Polygalacturonase; XET/XTH Xyloglucan endotransglucosylase/hydrolase. (**e** and **f**) The heat map illustrates the presence of AP2/ERF and bHLH/NAC cis regulatory elements in cell wall genes. *Purple* and *white* colors depict the presence and absence of the respective CRE in the promoter regions of the gene

In this study, more than 120 annotated cell wall (BIN 10) transcripts exhibited DE between *small* and *large* berries during fruit development (Additional file 1: Table S5). Transcripts related to cellulose synthesis (BIN 10.2), cell wall modification (BIN 10.7), degradation (BIN 10.6), and pectin esterification (BIN 10.8) were significantly modulated among the berry size classes in these clusters. A decrease in berry E has been shown to be the earliest ripening-associated event observed at the onset of ripening [13]. Emphasis will be given on the cell wall genes differentially expressed at 74 DAA (Fig. 6a–d), given that this is the developmental stage that immediately follows the large decrease in berry E and the stage when *small* and *large* berries displayed differences in E.

We observed that 17 cell wall degradation genes (BIN 10.6.2 and BIN 10.6.3), consisting of six xyloglucan endo-transglycosylase/trans-hydrolase, two 1,4-beta-mannan endohydrolase, five pectate lyases, and three polygalacturonases, were downregulated in skins of *small* compared to *large* berries at 74 DAA (Fig. 6a and b). This observation may reflect lower cell wall degradation activity in *small* than in *large* berries, which supports the slower rate of softening from 60 to 74 DAA. Pectin degrading enzymes, such as polygalacturonases and pectate lyases, are two principal enzymes involved in tomato fruit softening through active cell wall pectin de-polymerization [56]. Studies in tomato [60], apple [61], and strawberries [62] have demonstrated that silencing of polygalacturonase gene expression does not always affect fruit softening. Pose et al. [62] found large differences in strawberry fruit firmness at harvest in strawberry polygalacturonase 1-suppressed lines, but the changes in firmness in tomato during softening were very small [60] and in apple most transgenic polygalacturonase suppression lines had no effect on fruit firmness [61]. Nevertheless, silencing of pectate lyases transcripts result in firmer fruits both during softening and at harvest [63, 64].

In plants, the transcription factor APETALA2/Ethylene Responsive Factor (AP2/ERF) belongs to a multigenic family involved in the control of metabolism, stress response, and plant development through the binding of DRE and GCC-related motifs in promoters regions of regulatory targets (reviewed in [65]). Several studies in fruits have demonstrated that AP2/ERF TFs contribute in regulating ripening-related processes, especially fruit softening, by targeting cell wall degradation genes (reviewed in [48]). In this study, significant enrichment of ethylene related CREs, such as DRECRTCOREAT, CBFHV, and LTRECOREATCOR15 (Fig. 6e, Additional file 1: Table S4), in cluster 7 genes also corresponds to the presence of AP2/ERF CREs in promoter regions of seven (three 1,4-beta-mannan endohydrolase, two pectate lyases, and two polygaluacturonase) predicted cell wall degrading transcripts. In agreement, we observed eight DE AP2/ERF TFs which were significantly downregulated in *small* compared to *large* berries at 74 DAA; four of which were ripening-associated (Additional file 1: Table S3). This suggest that AP2/ERF TFs may be critical in regulating cell wall degrading transcripts in grapevines, and differences in AP2/ERF TF regulation between *small* compared to *large* berries prior to ripening, may have affected the progression of softening reaching this stage (Fig. 1d).

Expansins act as primary cell wall loosening agents and incorporators of new cell wall material during the first growth phase and as mediators of cell wall disassembly by facilitating the contact between cell wall material and degradation enzymes during stage III of growth in grapevine berries [59, 66]. The grapevine genome contains 29 expansin genes classified into four distinct subfamilies (expansin A, expansin B, expansin-like A, and expansin-like B) [67], of which two grapevine expansins were demonstrated to promote cell expansion [68]. Many cell wall expansin transcripts (BIN 10.7) were highly expressed at 74 DAA and six of these transcripts were significantly downregulated in skins of *small* with respect to *large* berries. Moreover, other six transcripts were downregulated at 121 DAA when their level of expression was on average low (Fig. 6c and d). The downregulation of expansin transcripts observed in the skins of *small* compared to *large* berries at 74 and 121 DAA indicates that cell wall loosening in the skin may be generally reduced, potentially limiting skin and mesocarp expansion and resulting in smaller berries. Indeed, fruits of tomatoes overexpressing expansin displayed enhanced softening and were significantly larger compared to controls at various stages of fruit development [69].

The regulation of some expansin transcripts such as *EXPA19* (VIT_18s0001g01130) and *EXPB4* (VIT_15s0021g02700) by VvCEB1 have also been proposed [55, 70]. Analysis of *VvCEB1*, a fruit ripening-specific bHLH transcription factor, gene expression from 10 different varieties exhibiting differences in berry size revealed a strong correlation between fruit size and *VvCEB1* transcript accumulation [71]. In this study, *VvCEB1* transcript (VIT_01s0244g00010) reached a maximum at 74 DAA and slowly decreased until 121 DAA (Additional file 1: Table S3). *VvCEB1* was downregulated in skins of *small* compared to *large* berries at 74 and 121 DAA, but were only significant at 121 DAA. The down regulation of *VvCEB1* in *small* berries at 74 and 121 DAA paralleled the down regulation of many expansin transcripts. Promoter analysis performed on DE expansins for bHLH-related CRE revealed that the CATGTG element, a typical DNA binding domain for bHLH TFs [72], was enriched in the promoter region of 10 out of  13 DE expansin genes (Fig. 6f, Additional file 1: Table S4). This suggests that upstream control of expansins differentially expressed in *small* and *large* berries may in part be mediated by VvCEB1 during ripening. Nevertheless, previous studies have also shown that NAC TFs can bind specifically to CATGTG elements [73], which might provide evidence for the regulation of expansin transcripts by NAC TFs. We observed that the majority of NACs, such as a ripening-associated *NAC26* (*VvNAC26*, VIT_01s0026g02710) which is highly expressed at 74 and 103 DAA, were downregulated in *small* compared to *large* berries at 74 DAA in parallel with downregulation of several expansin genes (Additional file 1: Table S3). A recent study has also shown that polymorphisms in *VvNAC26* were associated with berry size variation among 114 grapevine varieties [74]. Together, this transcriptional network involving bHLH and NAC TFs may be critical for regulating berry growth and size determination in grapevine.

### Modulation of flavonoid and stilbenoid pathways in *small* and *large* berries

**Fig. 7:**
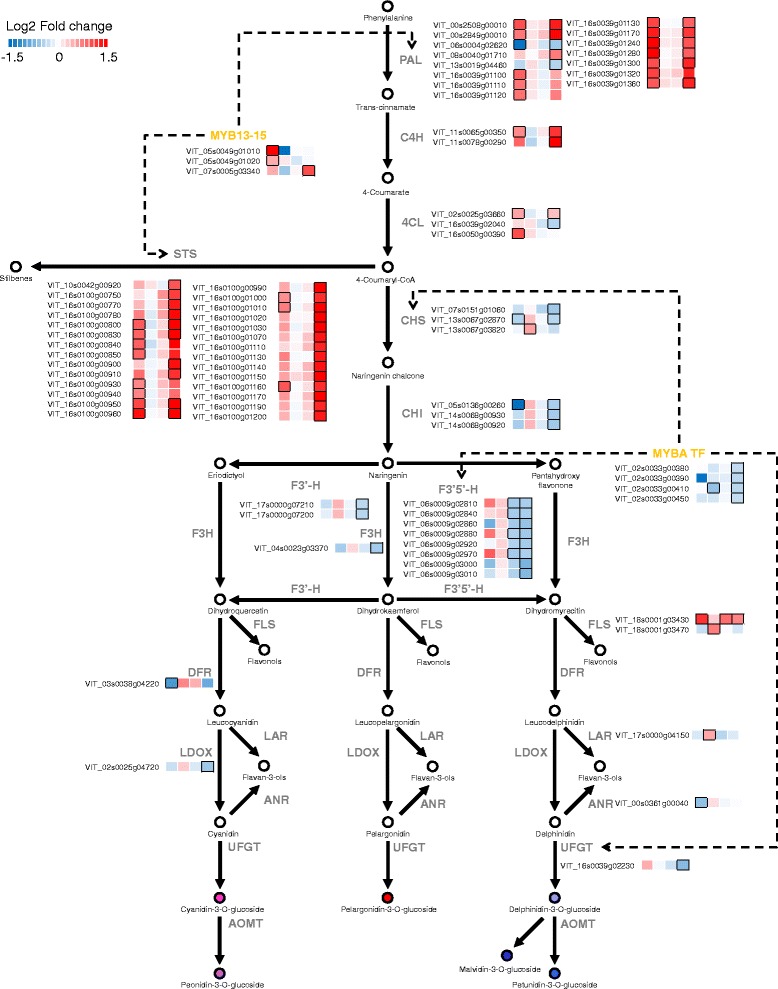
Modulation in berry skin transcripts involved in the phenylpropanoid and flavonoid pathway in *small* and *large* berries at 47, 74, 103, and 121 DAA. *Blue* and *red boxes* indicate downregulated and upregulated transcripts, respectively, in *small* berries in relation to *large* berries. Boxes with bold margins indicate significant differences (adjusted *P*-value <0.05) between berry size treatments at a given developmental stage. Transcription factors (colored in *yellow*) involved in the regulation of the phenylpropanoid and/or the flavonoid pathway transcripts are depicted in dotted lines. PAL Phenylalanine lyase; C4H Cinnamate-4-hydroxylase; 4CL 4-Coumarate:coenzyme A ligase; CHI Chalcone isomerase; CHS Chalcone synthase; F3H Flavanone 3-hydroxylase; F3′H Flavonoid 3′-hydroxylase; F3′5′H Flavonoid 3′5′-hydroxylase; FLS flavonol synthase; DFR Dihydroflavonol 4-reductase; LAR Leucoanthocyanin reductase; LDOX Leucoanthocyanin dioxygenase; UFGT UDPglucose:flavonoid 3-O-glucosyltransferase; and AOMT Anthocyanin O-methyltransferase. All other information is available at Additional file 1: Table S5

Flavonoids and stilbenes are plant secondary metabolites commonly found across the plant kingdom. These specialized metabolites fulfill diverse physiological roles in stress response, as antioxidants, and during reproduction [75]; in grapevine, they strongly affect grape and wine quality [76]. We observed that differences in berry size were related to large differences in the expression of flavonoid genes (Fig. 7, Additional file 1: Table S5). The DE flavonoid genes were predominantly assigned to clusters 1 (19 genes) and 6 (5 genes).

Three chalcone synthases (CHSs; VIT_07s0151g01060, VIT_13s0067g02870, and VIT_13s0067g03820), and three chalcone isomerases (CHIs; VIT_05s0136g00260, VIT_14s0068g00920, and VIT_14s0068g00930) -coding genes were differentially modulated in *small* and *large* berries. One *CHS* (VIT_13s0067g02870) and one *CHI* (VIT_05s0136g00260) were downregulated in *small* relative to *large* berries at 47 DAA when the pathway precursors are used for the production of flavan-3-ol and proanthocyanidins. Later during development, one *CHS* (VIT_13s0067g03820) was upregulated at 74 DAA, while two (VIT_07s0151g01060 and VIT_13s0067g02870) out of three CHSs and all the *CHI*s mentioned above were downregulated in *small* in comparison to *large* berries at 121 DAA.

Of the total 16 flavonoid 3′5′-hydroxylases (F3′5′Hs) found in the grapevine genome, we observed seven *F3′5′H* transcripts (VIT_06s0009g02810, VIT_06s0009g02840, VIT_06s0009g02860, VIT_06s0009g02880, VIT_06s0009g02920, VIT_06s0009g02970, and VIT_06s0009g03010) that were significantly downregulated in *small* berries at 103 and 121 DAA (Cluster 1). Similarly, two flavonoid 3′-hydroxylases (F3′Hs; VIT_17s0000g07200 and VIT_17s0000g07210) and one flavanone 3-hydroxylase (F3H; VIT_04s0023g03370) -coding genes, were also significantly downregulated in *small* berries at 121 DAA (Cluster 1). F3′5′H and F3′H enzymes have been shown to be critical in determining the accumulation of di- and tri-hydroxylated flavonoids and the ratio between blue and red anthocyanin in grapes [77–79]. The simultaneous downregulation of both *F3′H* and *F3′5′H* transcripts in *small* compared to *large* berries, particularly at 121 DAA, did not affect the relative abundance of the different anthocyanin forms (data not shown). Flavonol synthase (FLS) is the key enzyme for the production of flavonols in grapevines [80]. Of the five *FLS* transcripts encoded in the genome, the two transcriptionally active *FLS* (VIT_18s0001g03470 and VIT_18s0001g03430) exhibited a ripening-associated accumulation in the skin (Additional file 1: Table S5); similar to that observed in previous studies in developing grape berries [80, 81]. Transcripts encoding grapevine FLS5 (VIT_18s0001g03430) were significantly upregulated in *small* berries at 47, 103, and 121 DAA (Fig. 7). Similarly, the *FLS4* (VIT_18s0001g03470) gene was significantly upregulated in *small* with respect to *large* berries at 74 DAA. Previous studies have shown that *FLS* transcript expression provides an excellent indicator for cluster light exposure and marker for flavonol synthase activity given strong positive relationship (correlation) of solar radiation intensity with *FLS* transcripts and flavonol content in berry skins [82, 83]. In the current study, berries of different sizes were harvested from the same clusters controlling for differences in microclimate among berries. Thus differences in *FLS* expression between berries of different sizes must have arose from endogenous mechanisms. Consistently, previous studies reported that wine produced from small berries contain more flavonols compared to wines obtained from medium and large berries [24].

The expression level of key flavan-3-ol/proanthocyanidin genes codifying for leucoanthocyanidin reductase (LAR) and anthocyanidin reductase (ANR) were highly expressed at 47 DAA and decreased in the expression at 74 DAA, consistently with the early flavan-3-ols and proanthocyanidin accumulation during berry development [84]. Downregulation of *ANR* transcript at 47 DAA and upregulation of *LAR* transcripts at 74 DAA were observed in skins of *small* compared to *large* berries indicating a differential regulation of flavan-3-ol biosynthesis during stages that are critical for the accumulation of these compounds and their polymeric forms (proanthocyanidins) in the skin (Fig. 7).

Glycosylation of cyanidin and delphinidin via UDP glucose:flavonoid 3-O-glucosyltransferase (UFGT) [85] is critical in anthocyanin synthesis. The expression of grapevine *UFGT* (VIT_16s0039g02230) was significantly downregulated at 121 DAA (Cluster 1). MYBA1 and MYBA2 TFs redundantly regulates *UFGT* expression and modulates anthocyanin accumulation [86–88]. The *MYBA* transcripts were specifically activated at 74 DAA and decreases approaching 121 DAA (Fig. 7, Additional file 1: Table S5). *MYBA1* (VIT_02s0033g00410) was significantly downregulated in *small* compared to *large* berries at 74 and 121 DAA, while the others *MYBA*s were all downregulated at 121 DAA. The observed downregulation of grapevine *MYBA1* in *small* compared to *large* berries at 74 DAA is consistent with the lag of anthocyanin accumulation in smaller berries suggesting a delayed onset of ripening. Although no concurrent downregulation of *UFGT* with *MYBA1* was observed in *small* compared to *large* berries at 74 DAA, significant reduction of anthocyanin in *small* berries at 74 DAA might be strongly related to altered regulation of anthocyanin modification and transport pathways regulated by MYBA TFs [88]. Concurrent downregulation of *MYBA1*, *MYBA2*, and *MYBA3* with many flavonoid pathways transcripts (three *CHS*, seven *F3′5′H*, and *UFGT* transcripts) targeted by MYBAs [88] did not significantly reduce the concentration of anthocyanin in the skin at 121 DAA. Anthocyanin accumulation ceased from 103 DAA in both *small* and *large* berries. This indicates that the precursors for anthocyanin production (e.g. coumaroyl-CoA) are channeled to the production of other phenolic compounds, such as stilbenes, at late stages of ripening [89].

A marked upregulation of genes encoding enzymes involved in the general phenylpropanoid and stilbenoid pathway, namely phenylalanine ammonia lyase (PAL), cinnamic acid 4-hydroxylase (C4H), 4-coumarate:CoA ligase (4CL), and stilbene synthase (STS), was observed at 47 and 121 DAA (Fig. 7, Additional file 1: Table S5). Of particular interests are transcripts encoding STS enzymes. In plants producing stilbenes, STS catalyzes the production of cis- or trans-resveratrol using 4-coumaroyl-CoA and three molecules of malonyl-CoA. Interestingly, trends in gene expression of the majority of transcripts encoding STS and PAL were very similar (Additional file 1: Table S5). Several studies have reported that multiple STSs are responsible for the production of stilbene/resveratrol accumulation in the skin during berry development and in response to abiotic and biotic stress [89–91]. A large proportion of *PAL* and *STS* transcripts were significantly upregulated in *small* berries especially at 47 and 121 DAA (Fig. 7). Similar to that of anthocyanin, stilbenes largely accumulate at the onset of ripening [89–91]. However, no DE expression was observed at 74 DAA where their expression tends to peak. Concerted downregulation of *CHS* and upregulation of *STS* at 121 DAA in *small* compared to *large* berries might relieve the competition for coumaroyl-CoA and malonyl-CoA substrates by CHS; thereby favoring the flux to stilbene production, since anthocyanin levels have plateaued in both berries from 103 DAA onwards (Fig. 3d). Although the higher skin to berry weight ratio often determines a higher concentration of the anthocyanin and stilbenes accumulated in the skin [2, 3], concerted upregulation of the stilbene branch in *small* berries may enhance stilbene levels in wines made from small berries compared to normal and large sized berries harvested at the same time [24]. This induction of *PAL* and *STS* genes in *small* compared to *large* berries at 121 DAA might also be a consequence of altered hormone levels. Hormones such as ethylene and jasmonic acids are not only involved in the induction of key stilbene genes (*STS*) and levels in grapevines [92–94] but also, in the case of ethylene, might take part to the over ripening process [28].

Two grapevine MYB TFs, MYB14 (VIT_07s0005g03340) and MYB15 (VIT_05s0049g01020) directly regulate the biosynthesis of stilbene in grapevines [90]. In this study, *MYB15* had consistent expression trends, in both *small* and *large* berries, peaking at 74 DAA, while *MYB14* transcript evolution was not consistent between *small* and *large* berries. In *large* berries, *MYB14* transcripts progressively increase until 103 DAA before decreasing at 121 DAA; while, in *small* berries, *MYB14* expression peaked at 47 DAA before decreasing slightly at 74 DAA and increasing again later during ripening (Additional file 1: Table S5). Nonetheless, grapevine *MYB14* and *MYB15* were significantly upregulated in *small* berries at 121 and 47 DAA, respectively (Fig. 7). This result indicates their role in the regulation of *STS* expression and stilbene production in the skin.

### Modulation of fatty acid degradation pathways in *small* and *large* berries

**Fig. 8:**
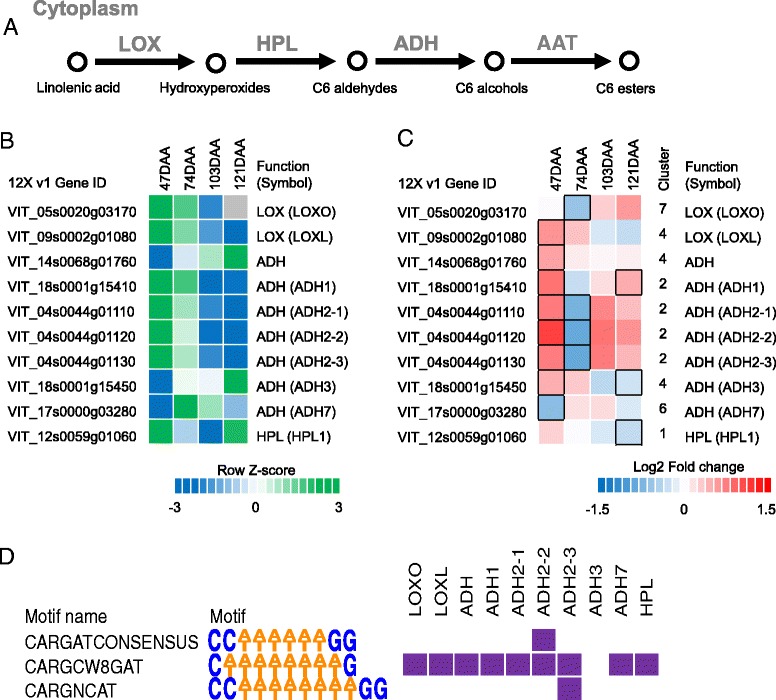
Berry skin transcript and selected cis-regulatory element profile of *small* and *large* berries of the fatty acid degradation/C6 volatile biosynthesis pathway at 47, 74, 103, and 121 DAA. (**a**) Simplified pathway schematic. The heat map represents the transcript evolution (**b**), based on the mean log2 (FPKM + 1) in *small* and *large* berries, and log2 fold (*small/large*) changes (**c**). The relative log2 (FPKM + 1) values from the four time points in **b** are depicted by *green* (high expression) and *blue* (low expression). *Grey* color indicates the absence (or low levels) of detectable transcripts at the corresponding stage. *Blue* and *red boxes* in **c** indicate downregulated and upregulated transcripts, respectively, in *small* berries in relation to *large* berries. Boxes with bold margins indicate significant differences (adjusted *P*-value <0.05) between *small* and *large* berries at a given developmental stage. The cluster column in **c** indicates the cluster the associated transcript belongs to. (**d**) The heat map illustrates the distribution of MADS box CREs in promoter regions of aroma-related transcripts differentially expressed between *small* and *large* berries. *Purple* and *white* colors depict the presence and absence of each CREs, respectively, in the promoter regions of the relevant transcripts. LOX Lipoxygenase; HPL Hydroperoxide lyase; ADH Alcohol dehydrogenase; AAT Alcohol acyl transferases

Aromatic alcohols and aldehydes derived from the fatty acid metabolism pathways (Fig. 8a), such as hexanol/hexenol and heptanal/hexanal, also contribute to late-ripening associated flavor/aromas in fruits and finished wines [95]. Several successive steps involving lipoxygenase (LOX), hydroperoxide lyase (HPL), alcohol dehydrogenases (ADHs), and alcohol acyl transferases (AATs) enzymes regulate the production of these compounds in plant tissues [96]. Many transcripts encoding enzymes of the aforementioned steps were modulated during fruit development and differed in the abundance between *small* and *large* berries (Fig. 8b and c, Additional file 1: Table S5). Lipoxygenase enzymes are involved in the formation of hydroperoxides from linolenic acid, which can lead to the accumulation of key wine aromas in the grape. Two *LOX* genes differentially expressed between *small* and *large* berries were highly expressed at 47 and 74 DAA before decreasing towards 121 DAA. One *LOX* transcript (VIT_05s0020g03170) was significantly downregulated at 74 DAA and another *LOX* transcript (VIT_09s0002g01080) was significantly upregulated at 47 DAA in *small* compared to *large* berries. Hydroperoxides are then converted to C6 related aldehydes, which often confer grassy-related aromas via HPL. One *HPL* transcript (VIT_12s0059g01060), mostly accumulated at 47 and 121 DAA, was downregulated in *small* compared to *large* berries. Subsequently, ADH converts C6 related aldehydes to C6 related alcohols, which can be further converted to produce volatile aromas via AATs. Interestingly, all seven *ADH* transcripts encoded in the grapevine genome were DE. Four *ADH* transcripts were highly expressed at 47 DAA and sharply decrease from 74 DAA and onwards; one *ADH* transcript peaked at 74 DAA while another two were highly expressed at 121 DAA (Fig. 8b). The four early developmental related *ADH* transcripts (VIT_18s0001g15410, VIT_04s0044g01110, VIT_04s0044g01120, and VIT_04s0044g01130), as well as two (VIT_18s0001g15450 and VIT_14s0068g01760) of the three late-ripening *ADH* genes, displayed significant upregulation in *small* compared to *large* berries at 47 DAA (Fig. 8c). On the contrary, at 121 DAA, one (VIT_18s0001g15450) of the two *ADH*s that are expressed at high levels at this stage was downregulated in *small* berries. The accumulation profiles of the different aldehydes, alcohols, and esters in Cabernet Sauvignon grapes during berry development and ripening has been reported and their formation is thought to involve a synergy between the biosynthesis and catabolism of various steps [97]. Although the molecular mechanisms are not fully understood, high levels of volatile esters in young berries (6 weeks post flowering) and high levels of alcohol compounds accumulated in later stages of berry development (12–14 weeks post flowering) are reported [97]. Therefore, the strong upregulation of four early ripening *ADH* and one *LOX* transcripts in *small* compared to *large* berries might favor a higher production of C6 aldehydes and subsequent turnover/flux to alcohols/esters (for fruitier aromas) during early development (47 DAA) in *small* compared to *large* berries.

In tomato, a MADS-box transcription factor (SlRIN) is a negative regulator of fruit ripening and acts as a modulator of aroma production in fruits via direct regulation of *ADH*, *LOX*, and *HPL* promoters [98]. We identified nine *MADS-box* TFs differentially expressed between *small* and *large* berries: four genes were allocated to clusters 2, two to clusters 6, and one to cluster 3, 5, and 7 individually. Of note are the predicted homologs of tomato *RIN* (VIT_14s0083g01050 and VIT_01s0011g00110), allocated into cluster 2, that share high expression similarity with four *ADH* transcripts from 47 to 121 DAA in *small* and *large* berries (Additional file 1: Table S5). Analysis of the promoter regions of the total DE fatty acid degradation pathway genes (two *LOX*, seven *ADH*s, and one *HPL*) for various MADS-box binding site (CArG box) revealed that one motif (CWWWWWWWWG) was located within all tested promoter regions except promoter region of an *ADH* transcript (Fig. 8d, Additional file 1: Table S4). These observations indicate that berry size may be associated with a different accumulation of aromatics in the fruit, likely through the coordinate regulation of MADS-box TFs and structural genes of the fatty acid degradation pathway.

## Conclusions

Significant differences in the physiology, biochemistry, and transcripts are found in different sized berries during fruit development. As *small* and *large* berries approach the onset of ripening, clear differences in the rate of development were apparent. Around the onset of ripening, the steeper drop in elasticity, more rapid accumulation of sugars, lower tartaric acid, and greater anthocyanin levels in *large* compared to *small* berries suggest that fruit size is associated to changes in the ripening physiology of the berry, where *large* berries approach ripening faster. These differences correspond to congruent changes in the hormonal pathways related to ABA, auxin, and ethylene via genes encoding NCED for ABA, TAA1/TAR and YUC for auxin, and ACO for ethylene. Genes encoding pathways contributing to fruit texture, flavor, and aroma were also differentially modulated accordingly to the berry size. The modulation of cell wall degradation and modification genes (e.g. PG and EXP) may contribute to the differences in elasticity decreases and berry growth/size. Upregulation of fatty acid degradation genes, especially *ADH*, during early development might favor production of desirable aromatics in *small* berries. In the late ripening stages, concurrent upregulation of phenylpropanoid and stilbenoid pathway genes with a parallel downregulation of the flavonoid pathway in skins of *small* compared to *large* berries indicates that smaller berries may have higher concentrations of aromatics and stilbenes, major antioxidants produced in the berry. Further investigation on endogenous and exogenous factors that regulate the fruit metabolism in berries of different sizes is necessary to identify the key factors – e.g. hormone signals, advantage of position within a cluster that favors mineral or water uptake, or a better microclimate – that determine the berry size itself as well as the transcriptome response. Finally, a deeper investigation of the fruit composition in relation to berry size may lead to the adoption of screening strategies based on size for tailoring fruit and wine quality.

## Additional file

Additional file 1: Table S1. Summary of RNA sequencing analysis metrics. RNA sequencing were carried in skins of *small* and *large* berries at four berry developmental stages namely 47 (before ripening, 4.9 °Brix), 74 (early ripening, 17.5 °Brix), 103 (ripening, 22.4 °Brix), and 121 (late ripening, 25.3 °Brix) days after anthesis (DAA). **Table S2.** Transcript abundance of the DE genes, reported as log2 (FPKM + 1) values, for each individual biological replicate (R1, R2, R3) in each treatment (*small* and *large* berry) at 47, 74, 103, and 121 days after anthesis. **Table S3.** Summary of differentially expressed genes between *small* and *large* berries at four berry developmental stages. All differentially expressed genes (Adj. *P*-value <0.05) and detailed description of the 12×V1 gene ID, log2 fold change values (*small* vs *large*), differential expression calls across four developmental stages, average log2 (FPKM + 1) values (3 replicates) of *small* and *large * berries together and individually, k-means assigned cluster (based on response and developmental stage), functional annotations based on Grimplet et al. [37] (including transcription factors), and MapMan pipeline. **Table S4.** Summary of PLACE- and PBM-curated cis-regulatory elements (CRE) analysis of k-means assigned clusters and selected group of genes. All information on the number of promoters with the specified CRE (match_in_sample), the number of genes within each group, number of promoters in the genome containing the specified CRE (match_in_genome), *P*-value and FDR of enriched CRE (FDR < 0.01), motif name and description, and a short description on selected CRE instances in groups. **Table S5.** Summary of selected hormone, cell wall, flavonoid/phenylpropanoid, and aroma/flavor metabolic pathways transcripts differentially expressed genes between *small* and *large* berries at four berry developmental stages which have been divided into separate classes and sub-categories. (XLSX 2418 kb)
